# Quercetin-Loaded Avocado Oil Nanoemulsion Reverses High-fat and High-carbohydrate Diet-Induced Testicular Dysfunction: Role of Oxidative Stress and NPY Signaling in a Randomized Trial

**DOI:** 10.5812/ijpr-165025

**Published:** 2025-12-31

**Authors:** Hamidreza Mazangi, Negar Panahi, Saeed Hesaraki, Shahabeddin Safi

**Affiliations:** 1 Department of Veterinary Basic Sciences, SR.C., Islamic Azad University, Tehran, Iran; 2 Department of Veterinary Pathobiology, SR.C., Islamic Azad University, Tehran, Iran

**Keywords:** Antioxidants, Male Infertility, Metabolic Disease, *Persea americana*, Quercetin

## Abstract

**Background:**

High-fat and high-carbohydrate diets (HF) can negatively affect male reproductive function, impairing spermatogenesis and testosterone production. Quercetin (Qu) may mitigate testicular damage, but its effectiveness is limited by poor bioavailability. Avocado oil (AO) improves blood lipid levels and enhances the absorption of fat-soluble nutrients.

**Objectives:**

We explore the impact of AO combined with quercetin on weight gain, dyslipidemia, hormonal dysregulation, testicular oxidative stress, and NPY signaling, all induced by a HF diet, to highlight testicular dysfunction and metabolic disorders.

**Methods:**

A nanoemulsion of avocado oil containing quercetin (NAOQu) was developed as an oil-in-water (O/W) nanoemulsion, utilizing the hydrophilic-lipophilic balance (HLB) value and conducting stability assessments, followed by characterization (DLS, Zeta potential, and TEM). Thirty rats were fed an HF diet for 8 weeks and treated with either Qu, a nanoemulsion of avocado oil (NAO), or NAOQu. Body weight was determined, and serum was analyzed for triglycerides (TG), cholesterol, HDL, and testosterone levels. Testicular homogenates were assessed for malondialdehyde (MDA), glutathione (GSH), superoxide dismutase (SOD) activity, and cholesterol. Hypothalamic NPY mRNA expression was quantified using qPCR.

**Results:**

The NAOQu (1% Qu, 1:1 avocado: Coconut oil) exhibited a stable, spherical morphology with a particle size of 286.7 nm, a zeta potential of -25.8 mV, and enhanced physical stability. The NAOQu significantly reduced body weight, improved lipid profiles (lowering TG and cholesterol while increasing HDL), and restored testosterone levels by 85% compared to high-fat diet (HFD) controls and reduced testicular MDA by 62% while increasing GSH and SOD activities by 2.3-fold and 1.8-fold, respectively. Molecular analyses revealed decreased testicular cholesterol levels and downregulated NPY mRNA expression, indicating reduced metabolic stress.

**Conclusions:**

The combined NAOQu formulation showed greater protective effects than Qu or AO alone, suggesting a synergistic effect. These findings highlight the potential of avocado oil-based nanoemulsion as an effective delivery system for Qu, offering a novel therapeutic strategy to counteract HF-induced male reproductive dysfunction and metabolic disease.

## 1. Background

Dietary habits greatly affect male reproductive health, especially a high-fat diet (HFD), which can lead to testicular dysfunction (1). Obesity and metabolic disorders caused by an HFD are linked to oxidative stress, inflammation, and hormonal imbalances. These factors can negatively affect spermatogenesis and testosterone production (2). The testis is particularly vulnerable to oxidative damage due to its high levels of polyunsaturated fatty acids and high metabolic activity. Unhealthy diets high in saturated and trans fats can harm sperm quality and fertilization rates, while a healthy diet can improve them. Additionally, sufficient intake of antioxidants plays a crucial role in preventing and treating male infertility (3). Obesity and diabetes negatively impact semen quality, sperm motility, and testosterone levels, indicating testicular dysfunction (4). An HFD can increase hypothalamic NPY, promoting feeding and reducing energy expenditure. Additionally, NPY inhibits the secretion of GnRH and LH, slowing gonadotropin release and reproductive processes during periods of low energy availability or metabolic stress (5). Possible underlying mechanisms include hormonal imbalances, oxidative stress, testicular dysfunction related to hypothalamic NPY signaling, and weight gain and metabolic disorders (6). Therefore, it is essential to identify therapeutic strategies to reduce testicular damage caused by an HFD to preserve male fertility.

Quercetin (Qu) is a dietary antioxidant with potential health benefits, including protection against osteoporosis, cancer, cardiovascular disease, and the effects of aging (7). It neutralizes harmful reactive species, contributing to its protective effects. Quercetin is recognized for its antioxidant, anti-inflammatory, and reproductive protective properties, but its low bioavailability limits its effectiveness (8). To improve absorption, new drug delivery systems (9) such as microemulsions have been developed (10).

Avocado oil (AO) contains beneficial compounds that may help manage diabetes and metabolic disorders by enhancing insulin sensitivity and reducing oxidative stress (11). While both AO and Qu have shown individual health benefits in preventing cardiovascular disease (12-14), there is limited research on their combined effects.

## 2. Objectives

We hypothesized that NAOQu would alleviate testicular dysfunction primarily by enhancing antioxidant delivery and modulating hypothalamic NPY signaling. This research aims to develop a stable nanoemulsion of avocado oil (NAO) containing quercetin to improve its efficacy, while also investigating the role of the NPY pathway in testicular function in rats. We assess the effects of NAOQu, Qu, and AO on serum lipid profiles, testosterone levels, testicular oxidative stress, and metabolic pathways.

## 3. Methods

### 3.1. Materials

The Qu powder was obtained from Sigma Aldrich. PEG 400, Tween 80, cholesterol powder, and sodium cholate were obtained from Merck. Egg yolk powder, corn starch, skimmed creamy milk, and lard were acquired from the local market. Pharmaceutical-grade, cold-pressed AO was obtained with a fatty acid composition of Oleic acid (18:1) 56%, Palmitic acid (16:0) 17%, Linoleic acid (18:2) 12%, Palmitoleic acid (16:1) 4.3%, Stearic acid (18:0) 0.2%, Linolenic acid (18:3) 0.5% and vitamin E 100mg/kg. All steps were approved by Dr. Faraz Mojab in the Department of Pharmacognosy, School of Pharmacy, Shaheed Beheshti University of Medical Sciences. Coconut oil was selected as a lipid vehicle with a different fatty acid profile (high in saturated MCTs) to distinguish the specific properties of AO's monounsaturated fats and bioactive composites from non-specific lipid effects.

### 3.2. Preparation of Quercetin-Loaded Avocado Oil-Based Nanoemulsion

The oil phase was prepared by dissolving 1 g of Qu in a mixture of 5 mL of AO and 5 mL of coconut oil (1:1) at 50°C. Tween 80 (15% v/v) and PEG 400 (5% v/v) were added as surfactant/cosurfactant, respectively, to formulate an oil-in-water (O/W) nanoemulsion based on the hydrophilic-lipophilic balance (HLB) value (15). To produce the NAO, all prior steps were performed without adding Qu in the oil phase. Coconut oil was included as a lipid vehicle control to optimize the HLB and enhance nanoemulsion stability at a low concentration (5% v/v). The mixture was stirred (600 rpm, 50°C, 30 min), then dispersed into 70 mL distilled water (50°C) under stirring (60 min) and sonication (200 W, 6 mm probe tip, 40 kHz, 10 min), shear rate during stirring (600 rpm), and criteria for stability assessment (visual observation for phase separation, turbidity measurement at 600 nm). The yellow color confirmed Qu incorporation and storage at 4°C (12).

### 3.3. Evaluation of Quercetin-Loaded Avocado Oil-Based Nanoemulsion

The vesicle size and zeta potential of the samples were measured using a Zetasizer Nano ZS (Malvern Instruments). First, the emulsion sample was diluted 100-fold with deionized water. The diluted sample was then injected into a disposable zeta cell (DT1060C) and the measuring chamber of the Malvern Zetasizer Nano ZS (UK) to evaluate the polydispersity Index (PDI). To assess the stability of the nanoemulsion and the nanoemulsion containing Qu, the prepared nanoemulsions were centrifuged at 3,000 rpm for 15 minutes to evaluate phase separation. A heating-cooling cycle was conducted by alternating between 40°C and 4°C, maintaining each temperature for 48 hours. This cycle was repeated three times. The formulation ratio of AO (5% v/v), coconut oil (5% v/v), Tween 80 (15% v/v), and PEG 400 (5% v/v), both with and without 1% Qu, was chosen based on earlier phase diagrams to facilitate the development of stable nanoemulsions. Stable nanoemulsions, with and without 1% Qu, were kept at 4°C for 30 days to observe any changes in particle size and zeta potential (less than 10% variation).

### 3.4. Examination of Avocado Nanoemulsions Containing Quercetin by TEM

The morphology of the nanoemulsion was examined using a transmission electron microscope (TEM; JEM-1200EX, Japan). The nanoemulsion was diluted 1:50 with deionized water and gently mixed for TEM analysis. A drop of the diluted sample was placed onto a holey film grid, stained with one drop of 2% aqueous phosphotungstic acid, and allowed to dry before imaging under electron microscopy.

### 3.5. Experimental Method

This study was conducted on 30 adult male Wistar rats weighing approximately 200 - 250 grams. The animals were obtained from the Pasteur Laboratory Animal Breeding Center and housed in standard cages for a week to acclimate before undergoing eight weeks of treatment (22 ± 2°C, 55 ± 10% humidity, 12-hour light/dark cycle). The experiment adhered to NIH guidelines and received ethical approval from the Research Ethics Committee of Islamic Azad University Science and Research Branch, with approval ID (IR.IAU.SRB.REC.1401.163). The Wistar rats were randomly divided into five groups and housed in a controlled environment with a 12-hour light/dark cycle. A high-fat and high-carbohydrate diet (HF) was formulated, containing 10% lard, 5% egg yolk powder, 2% cholesterol powder, 4.5% skimmed creamy milk, 0.5% sodium cholate, 5% sugar, and 33% cornstarch. It was added to standard rat chow (13). Twenty-four rats were fed a high-fat, high-sugar diet ad libitum for eight weeks to induce obesity and fatty liver disease, while six rats received a regular diet as the healthy group (Group H). The rats were randomly divided into five groups of six, as follows:

-Group H (healthy)

-Group HF (received the HF diet)

-Group HFD+NAO (received avocado oil nanoemulsion, 0.5 mL/day)

-Group HFD+Qu (received Qu 1% per day)

-Group HFD+NAOQu (received avocado oil nanoemulsion containing Qu 1%, 0.5 mL/day)

The 1% quercetin dose (25 mg/kg/day) was selected based on previous efficacy studies in rat models (7) and nanoemulsion stability. Throughout the study, the animals were weighed every week. The evaluation of obesity development was conducted through consistent monitoring of body weight. After the eight-week evaluation, all groups underwent a 12-hour food restriction, followed by a 48-hour observation period after the last drug dose was administered by gavage. While under deep anesthesia with ketamine and xylazine, the animals were euthanized. All biochemical tests were conducted using serum samples or testicular homogenates. Every step was carried out in duplicate, following the manufacturer's guidelines.

### 3.6. Serum Lipid Profile and Testosterone Levels

The serum was collected to analyze HDL, triglyceride (TG), cholesterol, and testosterone levels. This process was conducted under anesthesia using a combination of ketamine and xylazine. The serum samples were then stored at -20°C in a freezer for measurement using an automated biochemical analyzer (Mindray BS-200, China) with commercially available enzymatic reagent kits (Pars Azmun, Iran) (14). Testosterone levels were quantified using a commercial solid-phase competitive ELISA kit (Diagnostics Biochem Canada Inc., Canada, CAN-TE-250). The assay was performed on a BioTek Synergy H1 microplate reader (Agilent, USA) at 450 nm with a reference wavelength of 630 nm.

### 3.7. Testicular Tissue Evaluation Parameters

#### 3.7.1. Testicular Cholesterol Levels

Fresh testicular tissue (100 mg) was homogenized in 1 mL ice-cold PBS-EDTA using an ultrasonic homogenizer (20 kHz, 3 × 10 sec pulses on ice). Homogenates were centrifuged at 10,000 g (4°C, 15 min), and supernatants were collected. Testicular tissue cholesterol was extracted and quantified using a commercial colorimetric assay kit (Pars Azmun, Iran, Cat. CH-01) based on the cholesterol oxidase method (14).

#### 3.7.2. Measurement of Oxidative Stress Biomarkers (Malondialdehyde, Glutathione, Superoxide Dismutase)

Testicular tissue was isolated on ice. Tissue homogenization was performed in PBS containing EDTA and protease inhibitors using an ultrasonic homogenizer. The supernatant was mixed with thiobarbituric acid (TBA) and the trichloroacetic acid assay kit (Cayman Chemical, USA). Total glutathione (GSH) content was determined using a colorimetric assay kit (ZellBio GmbH, Germany). Superoxide dismutase (SOD) enzyme activity was measured using a tetrazolium salt-based colorimetric assay kit (ZellBio GmbH, Germany)

### 3.8. NPY mRNA Gene Expression

Snap-frozen brain tissue (50 mg) was homogenized in 1 mL TRIzol^®^ using a bead homogenizer (30 sec, 4°C). Total RNA was isolated using the QIAGEN RNeasy Plus Mini Kit according to the manufacturer’s protocol. 10 µL SYBR Green Master Mix, 1 µL cDNA, and 0.5 µM each primer (Table 1) was used for qPCR. The SYBR Green Master Mix Kit (Parstous, Iran) was used for real-time PCR (5). Relative NPY mRNA expression was calculated using the 2^-ΔΔCt^ method and expressed as a fold change compared to the H group.

**Table 1.** NPY Primers

Primers	Forward	Reverse
**NPY **	5' CCCAGAGCAGAGCACC 3'	5’ AGCAGGGATAGAGCGAG 3’
**β-actin**	5' CCATCTATGAGGGTTACGC 3'	5’ TGTAGCCACGCTCGGTC 3’

### 3.9. Statistical Analysis

Statistical analyses were performed using GraphPad Prism version 10.4.1. Data are presented as the mean ± standard error (SEM). For the analysis of NPY mRNA gene expression data, values are presented as mean ± standard deviation (SD). All datasets were tested for normality (Shapiro-Wilk test) and homogeneity of variances (Levene's test). A one-way ANOVA followed by Tukey's post-hoc test was used for comparisons across groups. The effect size for ANOVA is reported as eta-squared (η²). A P-value < 0.05 was considered statistically significant.

## 4. Results

### 4.1. Particle Size and the Zeta Potential of Globules

The globule size of the nanoemulsion, which consisted of 5% AO, 5% coconut oil, 5% polyethylene glycol 400, and 15% Tween 80, was measured at 100.43 nm. In contrast, the particle size of the avocado nanoemulsion containing Qu was 286.7 ± 24.9 nm. Additionally, the zeta potential of the avocado nanoemulsion formula containing Qu was -25.8 mV, compared to -16.7 mV for the avocado nanoemulsion formulation (Table 2). The HLB of the surfactant (14.4) significantly exceeds the required HLB for the oil phase (6.0). The 1:1 avocado: Coconut oil ratio with 1% Qu loading exhibited optimal characteristics, including minimal phase separation after centrifugation at 3,000 rpm for 15 minutes and 30-day stability at 4°C. This delivered a 4.1 mg/0.5 mL dose, equivalent to 20 mg/kg/day, accounting for nanoemulsion-enhanced bioavailability. Higher concentrations (1.5 - 2%) resulted in increased particle size (from 286.7 ± 24.9 nm to 412.5 ± 38.6 nm) and reduced zeta potential stability (from -25.8 mV to -10.3 mV).

**Table 2.** Globule Size, Zeta Potential, and Electrophoretic Mobility of the Optimal Oil-Water Emulsions with Quercetin Formulation ^a^

Variables	Globule Size (nm)	Zeta Potential (mV)	PDI	Surfactant HLB
**Avocado oil-water emulsions **	100.43 ± 10.2	-16.7	0.3019	14.4
**Avocado oil-water emulsions with QU**	286.7 ± 24.9	-25.8	0.377	14.375

Abbreviations: PDI, polydispersity Index; HLB, hydrophilic-lipophilic balance; QU, quercetin.

^a^Values are expressed as mean ± SD

### 4.2. Image of a Vesicle Under a Transmission Electron Microscope

**Figure 1.**
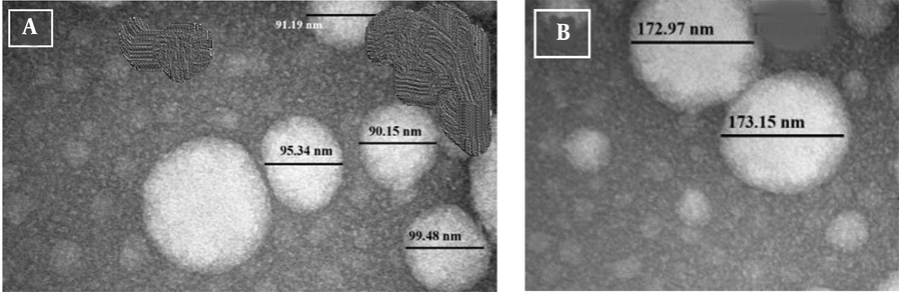
Morphology of nanoemulsion by TEM image, avocado oil-water emulsions (A); avocado oil-water emulsions with quercetin (Qu) (B).

The TEM image of the vesicle appeared spherical, with particles approximately 90 nm in diameter for Avocado oil-water emulsions (Figure 1A), and 170 nm in diameter for Avocado oil-water emulsions with quercetin (Figure 1B), demonstrating the spherical form of the nanoemulsion droplets and consistent with the O/W structure. The PDI of the emulsion, approximately 0.3, indicated the homogeneity and stability of the droplet size in the emulsion. The oil-water nanoemulsion (1, 3, 1, o/s/co) was found to be stable.

### 4.3. Body Weight

**Figure 2.**
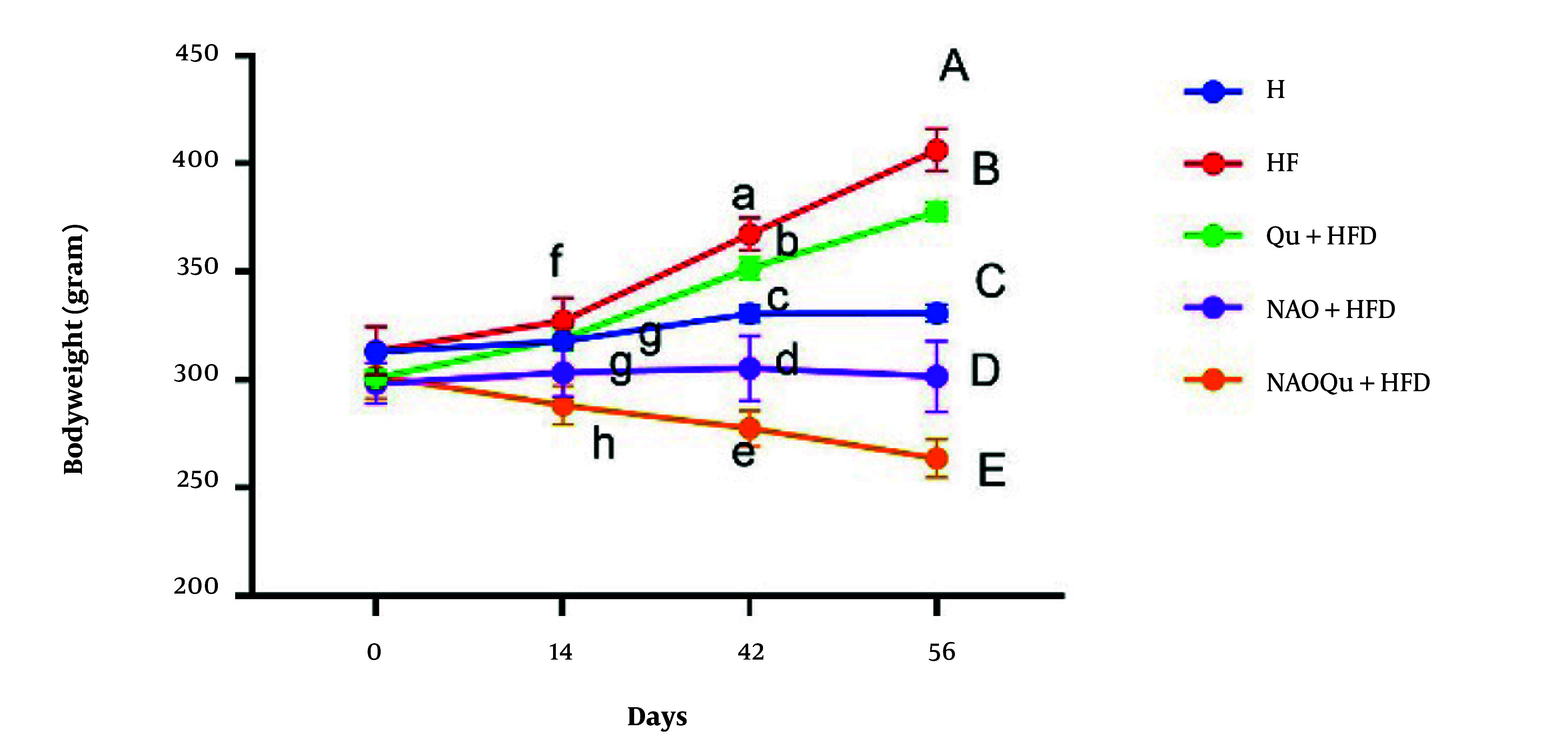
Body weight over 8 weeks (W0, W2, W6, W8). The groups include a healthy control (H), a high-fat, high-sugar diet group (HF), and three treatment groups [nanoemulsion of avocado oil (NAO), quercetin (Qu), and nanoemulsion of avocado oil containing quercetin (NAOQu)], which also receive a high-fat, high-sugar diet (n = 6). Data are expressed as the mean ± standard error of the mean (SEM). Letters f, g, and h indicate significant differences between groups at 14 days. Letters a, b, c, d, and e indicate significant differences between groups at 42 days. Letters A, B, C, D, and E indicate significant differences between groups at 56 days. Interaction term in the two-way repeated measures (RM) ANOVA [F (12, 75) = 142.7, P < 0.0001] with Tukey’s post-hoc test for the final time point.

In the NAOQu+HFD group, significant weight loss was observed compared to the HF group [mean diff. = 60.50, 95% CI (50.69, 70.31), P < 0.0001]. The HF group showed a significant difference compared with the H group (P < 0.0001; mean difference = -50.67). Weight loss was observed in both the NAO and NAOQu groups, with significant differences observed compared to the H group (P = 0.0017 for NAO and P < 0.0001 for NAOQu). Separate administration of Qu and avocado oil resulted in weight loss, with avocado oil-water nanoemulsions leading to a greater reduction than Qu alone (P = 0.0003) (Figure 2). 

### 4.4. Lipid Profile

HDL levels in the HF group were significantly lower than the H group [mean diff. = -9.33 mg/dL, 95% CI (-15.16, -3.51), P = 0.0072]. In the Qu+HFD group, HDL levels were higher than in the HF group, and the NAO+HFD group also showed higher HDL levels; however, this increase was not statistically significant. A significant increase in the NAOQu+HFD group was observed (P < 0.0001).

As shown in Table 3, post-hoc analysis confirmed that the HF successfully induced dyslipidemia, with significantly higher TG levels compared to the H group [mean diff. = 85.83 mg/dL, 95% CI (76.37, 95.29), P < 0.0001]. In the Qu+HFD and NAO+HFD groups, TG levels decreased compared to the HF group (P < 0.0001 and P = 0.0003, respectively). Furthermore, the NAOQu+HFD group demonstrated a significant reduction in TG compared to the HF group (P < 0.0001).

The cholesterol level in the H group was approximately 85 mg/dL, whereas it increased to 168 mg/dL in the HF group [mean diff. = -82.17 mg/dL, 95% CI (-94.66, -69.68), P < 0.0001]. In the Qu+HFD group, cholesterol levels decreased to approximately 116 mg/dL, compared to the HF group (P = 0.0001). The NAOQu+HFD treatment group recorded an average cholesterol level of about 95 mg/dL. In the NAO+HF group, the average cholesterol level was approximately 106 mg/dL (Table 3). 

**Table 3:** . Serum Lipid Profile ^a, b, c, d^

Variables (mg/dL)	H	HF	Qu+HF	NAO+HF	NAOQu+HF
**Serum triglycerides **	82.67 ± 0.8 ^A^	168.5 ± 2.4 ^B^	118.3 ± 1.3 ^C^	121.5 ± 1.8 ^C^	108.0 ± 1.9 ^D^
**Serum total cholesterol**	83.50 ± 1.7 ^A^	165.7 ± 2.6^B^	121.2 ± 1.5 ^C^	133.2 ± 1.3 ^D^	98.00 ± 2.05 ^E^
**Serum HDL **	35 ± 0.9 ^A^	25.67 ± 1.08 ^B^	30 ± 0.6 ^B^	33.67 ± 0.5 ^A^	44.83 ± 0.6 ^C^

Abbreviations: H, healthy control; HF, high-fat, high-sugar diet group; NAO, avocado oil; Qu, quercetin; NAOQu, nanoemulsion of avocado oil containing quercetin.

^a^ The groups include a healthy control (H), a high-fat, high-sugar diet group (HF), and three treatment groups (NAO, Qu, and NAOQu) that also receive a high-fat, high-sugar diet (n = 6).

^b^ Values are expressed as the mean ± standard error of the mean (SEM).

^c^ Superscript letters A, B, C, D, and E indicate significant differences between groups.

^d^ Statistically significant effects of treatment on serum triglyceride levels [F (1.94, 9.70) = 318.5, P < 0.0001, η² = 0.985], total cholesterol [F (2.36, 11.79) = 262.3, P < 0.0001, η² = 0.981], and HDL levels [F (2.19, 10.93) = 78.24, P < 0.0001, η² = 0.940].

### 4.5. Testosterone Levels

**Figure 3.**
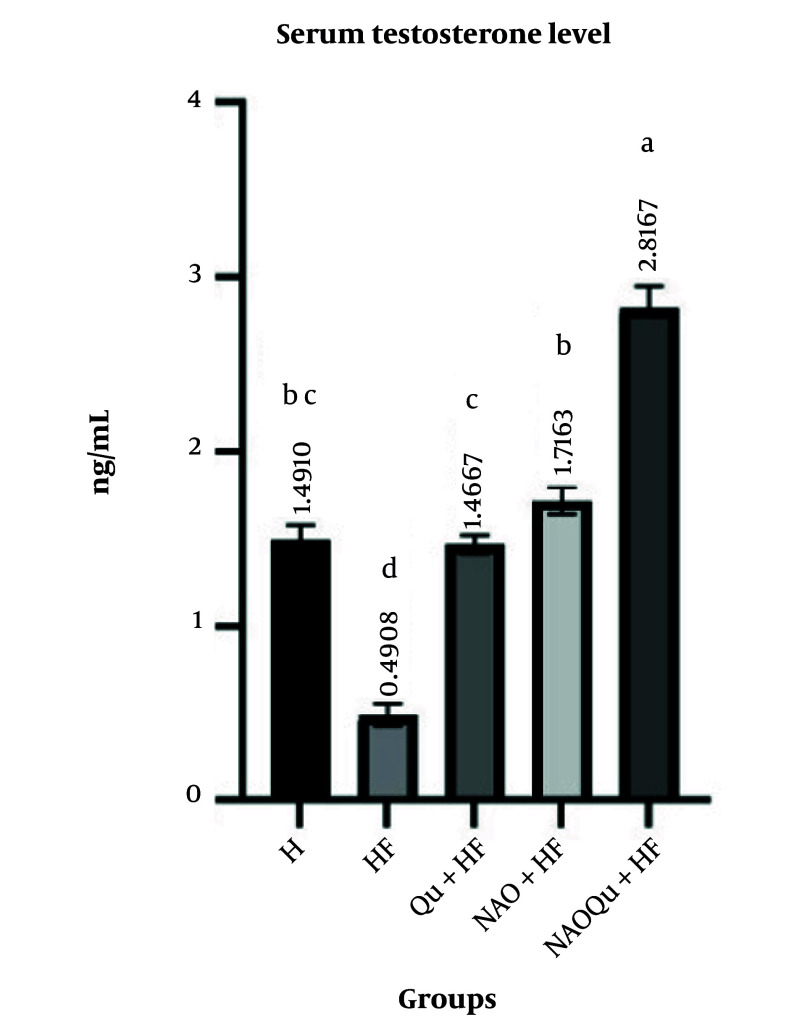
Serum testosterone level. The groups include a healthy control (H), a high-fat, high-sugar diet group (HF), and three treatment groups [nanoemulsion of avocado oil (NAO), quercetin (Qu), and nanoemulsion of avocado oil containing quercetin (NAOQu)] that also receive a high-fat, high-sugar diet (n = 6). Data are expressed as the mean ± standard error of the mean (SEM). Letters a, b, c, d, and e indicate significant differences between groups. [F (1.752, 8.759) = 97.2, P < 0.0001, η² = 0.951], indicating a very large effect size.

The testosterone levels decreased significantly in the HFD group compared to the H group (P < 0.0005, Figure 3). Post-hoc pairwise comparisons with Bonferroni correction revealed that the NAOQu treatment significantly increased testosterone levels compared to the HFD group [mean difference = 4.15, 95% CI (3.19, 5.11), P < .001, d = 5.27], and this effect was significantly greater than both the Qu [mean difference = 1.89, 95% CI (0.93, 2.85), P = .001, d = 2.40] and avocado-oil-nanoemulsion groups [mean difference = 2.25, 95% CI (1.29, 3.21), P < .001, d = 2.86].

### 4.6. Testicular Antioxidant and Oxidant Levels 

**Figure 4.**
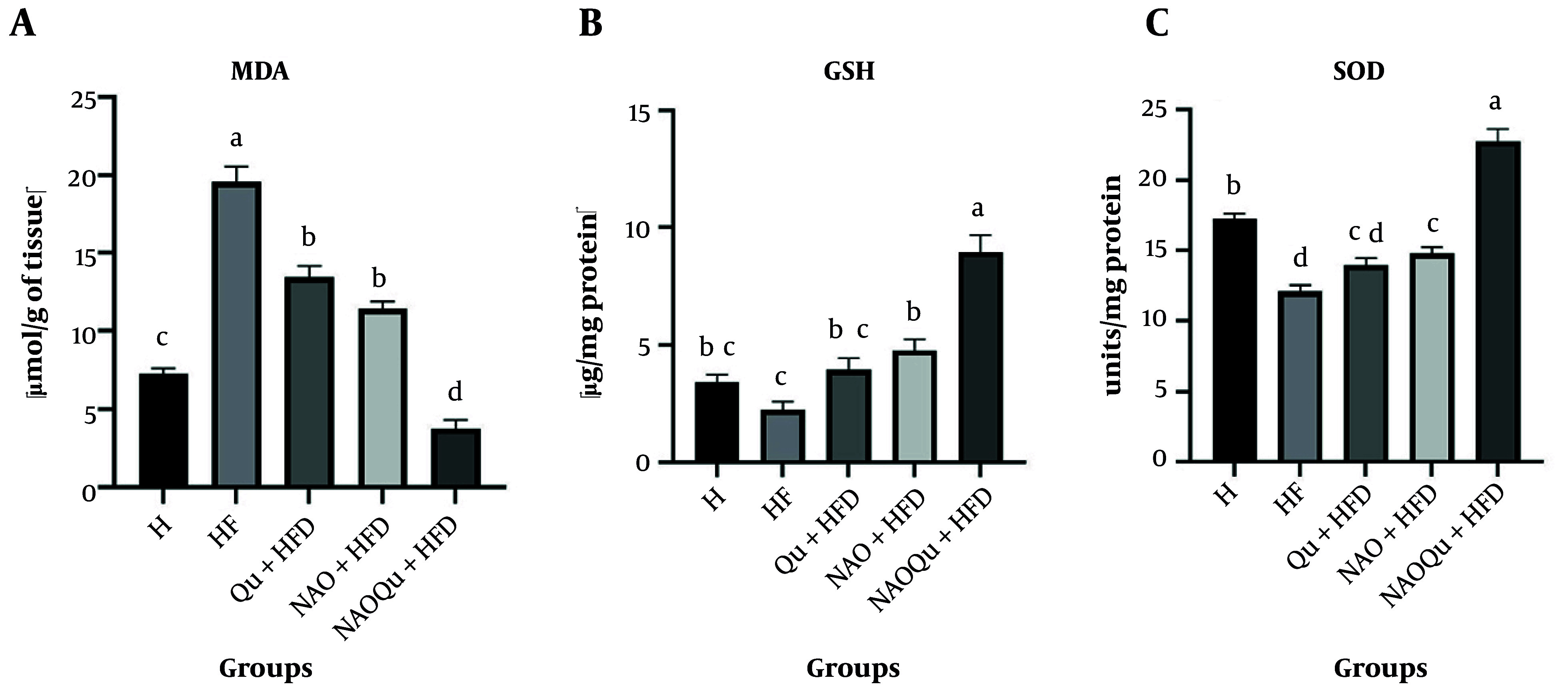
Testicular malondialdehyde (MDA) (A); and increasing glutathione (GSH) (B); and superoxide dismutase (SOD) (C); activity. The groups include a healthy control (H), a high-fat, high-sugar diet group (HF), and three treatment groups [nanoemulsion of avocado oil (NAO), quercetin (Qu), and nanoemulsion of avocado oil containing quercetin (NAOQu)] that also receive a high-fat, high-sugar diet (n = 6). Data are expressed as the mean ± standard error of the mean (SEM). Letters a, b, c, and d indicate significant differences between groups. Testicular MDA levels, a marker of lipid peroxidation [F (4, 25) = 89.03, P < 0.0001, η² = 0.934], GSH levels [F (4, 25) = 27.09, P < 0.0001, η² = 0.813] and SOD activity [F (4, 25) = 56.02, P < 0.0001, η² = 0.900].

The HF group showed a significant difference in malondialdehyde (MDA) level (P < 0.0001). A combination of AO and Qu significantly decreased MDA levels (P < 0.0001). No significant difference was observed between the NAOQU and H groups (P = 0.9996) (Figure 4A). The NAOQu exhibited the most significant increase in GSH levels (Figure 4B) compared to the HF and H groups (P < 0.0001). In the HF group, SOD activity was significantly lower than in the H group (P < 0.0001). In contrast, a notable increase in SOD activity was observed (Figure 4C) with the administration of a combination of AO and Qu (P < 0.0001).

### 4.7. Testicular Cholesterol Levels

**Figure 5.**
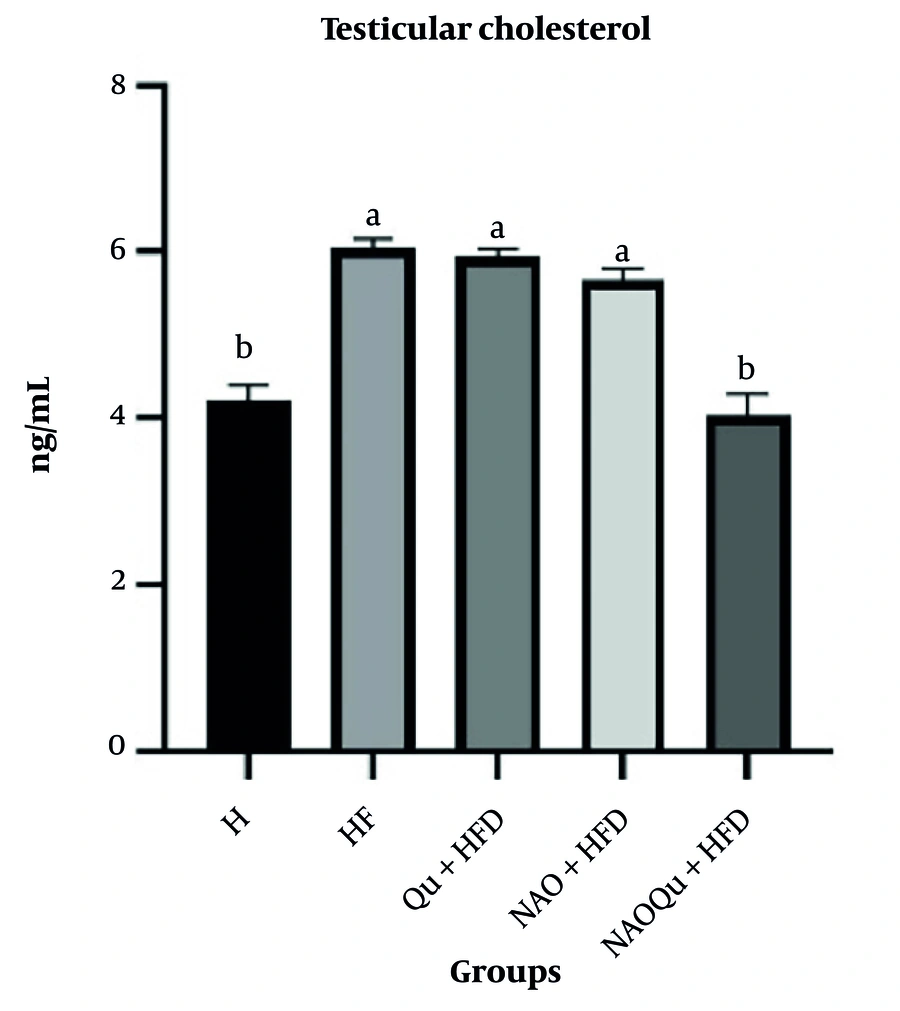
Testicular Cholesterol Levels. The groups include a healthy control (H), a high-fat, high-sugar diet group (HF), and three treatment groups [nanoemulsion of avocado oil (NAO), quercetin (Qu), and nanoemulsion of avocado oil containing quercetin (NAOQu)] that also receive a high-fat, high-sugar diet (n = 6). Data are expressed as the mean ± standard error of the mean (SEM). Letters a, b, c, and d indicate significant differences between groups [F (4, 30) = 32.91, P < 0.0001, η² = 0.814].

Administration of an HF diet significantly increased testicular cholesterol (P < 0.0001) with no significant difference observed between the Qu and avocado nanoemulsion groups (Figure 5). Avocado administration significantly decreased cholesterol levels in the testes compared with the HF group (P < 0.0001). No significant difference was observed between H and NAOQU groups (P = 0.9440).

### 4.8. NPY mRNA Gene Expression

**Figure 6.**
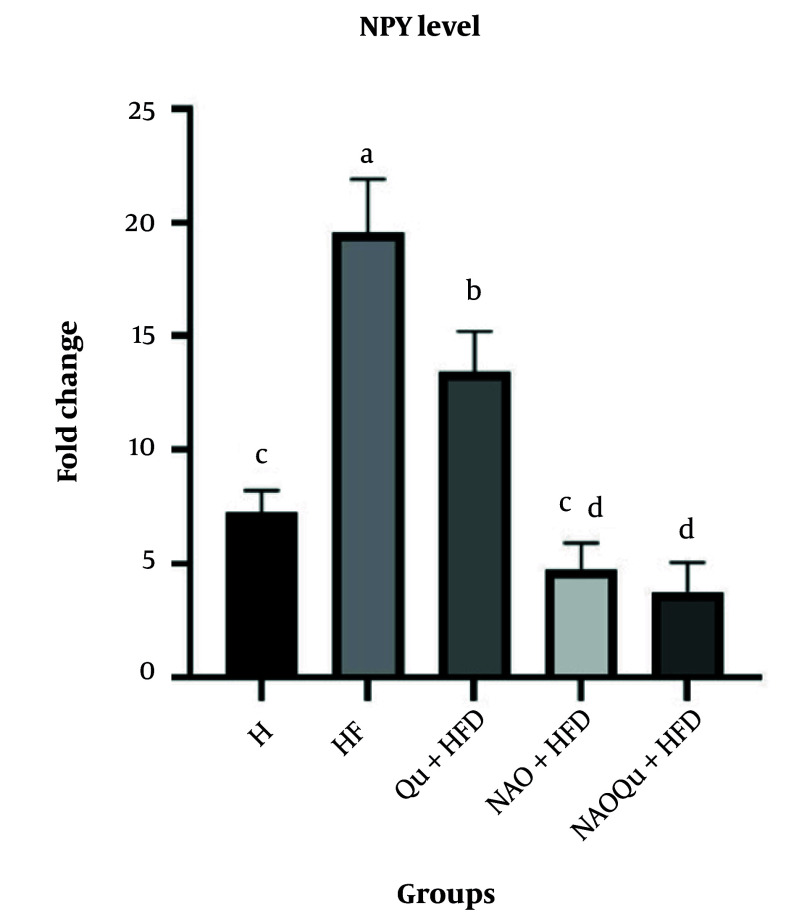
The NPY mRNA gene expression. The groups include a healthy control (H), a high-fat, high-sugar diet group (HF), and three treatment groups [nanoemulsion of avocado oil (NAO), quercetin (Qu), and nanoemulsion of avocado oil containing quercetin (NAOQu)] that also receive a high-fat, high-sugar diet (n = 6). Data are expressed as the mean ± standard deviation (SD). Letters a, b, c, d, and e indicate significant differences between groups. Analysis performed using one-way ANOVA and Tukey’s post-hoc test [F (4, 25) = 106.7, P < 0.0001, η² = 0.945].

The HF group exhibited the highest NPY mRNA gene expression (Figure 6), consistent with the weight gain (P < 0.0001). Quercetin consumption moderately reduced this gene expression level, with the most significant reduction observed in the nanoemulsion of AO and NAOQU consumption (P < 0.0001). Notably, the intake of Qu-loaded AO in the NAOQu group also significantly decreased the mRNA level compared to the H group [mean diff. = 3.46, 95% CI (0.79, 6.14), P = 0.0067].

## 5. Discussion

The 100-nm particle size and -16.7 mV zeta potential of the nanoemulsion, along with the 286.7 nm particle size and -25.7 mV zeta potential of NAOQu, enhance Qu's solubility and testicular uptake, thereby addressing its poor bioavailability (approximately 4%). These advancements in nutraceutical nano-delivery help improve Qu's therapeutic use, which is often limited by its low water solubility. Oil-water emulsions can further enhance their solubility and stability for medical and food applications (16). The spherical morphology observed by TEM further supports uniform dispersion and cellular uptake efficiency, consistent with previous studies on lipid-based nanoformulations (17). The measured difference between the DLS-determined hydrodynamic diameter (286.7 ± 24.9 nm) and the TEM-determined core diameter (approximately 170 nm) arises because DLS assesses particle size in suspension, which includes the core, the surfactant layer (Tween 80/PEG 400), and the solvation shell. In contrast, TEM measures the dry core diameter in high vacuum, excluding the hydration layer and possibly causing the surfactant coating to collapse. This anticipated variation underscores the presence of a significant hydrophilic corona surrounding the oil core, which is crucial to the nanoemulsion's stability and biological function. The HLB of the surfactant (14.4) significantly exceeds the required HLB for the oil phase (6.0). This suggests that the current formulation is overly hydrophilic and is likely to promote O/W nanoemulsions. The formulation was refined by matching the HLB value and conducting stability assessments.

Initial in vivo pharmacokinetic studies demonstrate the oral bioavailability and tissue distribution of polyols (avocadene and avocadyne), highlighting their promise as bioactive delivery agents and co-surfactants to enhance the encapsulation of poorly water-soluble medications (18). Quercetin is poorly water-soluble and lipophilic, which necessitates a system that improves its solubility and bioavailability (19). The HFD group showed increased MDA levels and decreased GSH and SOD levels, indicating oxidative damage linked to obesity (20). Elevated testicular cholesterol in HFD rats was associated with sperm membrane dysfunction (21). Quercetin's antioxidant properties, enhanced by nanoemulsion delivery, likely reduced lipid peroxidation and increased endogenous antioxidants (22). The NAOQu effectively reversed HFD-induced hyperlipidemia, lowering TG and cholesterol to baseline levels, possibly through improved intestinal absorption via nanoemulsion carriers (9). Our results were consistent with studies showing that polyphenol-rich diets protect against spermatogenic dysfunction, reduce estrogen conversion, and that AO activates PPAR-α (11), which enhances fatty acid oxidation (12, 18, 23). This aligns with the known anti-adipogenic effects of QU and the role of AO in lipid metabolism (24).

The NAOQu restored testosterone levels and testicular cholesterol to healthy levels, outperforming Q or AO alone. This highlights NAOQu’s dual role in mitigating HFD-induced hypogonadism and lipid dysregulation, possibly through modulation of the NF-κB/Nrf2 pathway (7). The NAOQu also reversed HFD-induced dyslipidemia and weight gain, suggesting that it targets pathways such as PPAR-γ and AMPK (25), which are critical in lipid metabolism (26). Our study showed that NAOQu reduced cholesterol accumulation, potentially by modulating SREBP through Qu (27) and AO’s lipid-clearing effects (18). Additionally, NPY mRNA overexpression (attributable to obesity-driven hyperphagia) was suppressed by NAOQu, suggesting a central metabolic benefit (28). Moreover, AO induced significant weight loss in HFD-fed groups, possibly by suppressing NPY mRNA, which regulates appetite and adiposity (5). Therefore, it can be concluded that the combination of AO and Qu as a synergistic treatment contributed to weight loss. Diet-induced obesity can cause neuropathy of NPY+axons (29). Furthermore, modulation of NPY signaling has been directly linked to the amelioration of HFD-induced reproductive dysfunction (30), as demonstrated by improvements in reproductive parameters following NPY2R-targeted treatment in a recent study. This provides a strong, contemporary logical link for how NAOQu-induced modulation of the NPY pathway could contribute to the restoration of testicular function in our model (5). Further research incorporating protein-level validation methods (such as Western blotting and immunohistochemistry) is necessary to verify the involvement indicated by NPY mRNA downregulation, and pharmacokinetic and biodistribution studies are essential to assess quercetin uptake in the testes and to correlate findings with direct fertility measures, including sperm parameters.

This study established AOQ as a new, multifaceted therapeutic strategy for addressing metabolic and reproductive dysfunction caused by HF. The nanoemulsion platform overcomes Qu’s bioavailability limitations by leveraging the antioxidant synergy of AO to mitigate oxidative stress (MDA, SOD, GSH) and lipid profiles, restore testosterone levels and testicular cholesterol homeostasis, and promote weight management through modulation of NPY mRNA. This study has limitations, including a small sample size and a lack of direct assessment of fertility using sperm parameters such as count and motility. Future research should include pharmacokinetic analysis to confirm the enhanced bioavailability of quercetin from the NAOQu formulation.

Our study demonstrated that quercetin-loaded avocado nanoemulsion effectively mitigates HF-induced testicular dysfunction and metabolic disease by improving body weight and lipid metabolism, reducing oxidative stress, and restoring testosterone levels in Wistar rats. The combined formulation, NAOQu, demonstrated better performance than either quercetin or the NAO, indicating a synergistic therapeutic effect.

## Data Availability

The data presented in the study are included in the article, and also available on request from the corresponding author during submission or after publication.
